# Supplementary material for: Human-robot interaction: the impact of robotic aesthetics on anticipated human trust
Source: PeerJ Comput Sci. 2022 Jan 14;8:e837. doi: 10.7717/peerj-cs.837 (PMC8771782; doi:10.7717/peerj-cs.837)
Supplement: Supplemental Information 1 [file peerj-cs-08-837-s001.docx]

A study to investigate the impact of the aesthetic on people’s trust of robots. Published

Survey Flow

Block: Participant information/ Introduction/ Happy Face (17 Questions)

Standard: Human Eyes (5 Questions)

Standard: Confused face (5 Questions)

Standard: Colour Changes (6 Questions)

Standard: Tester Pick Red / Contrast images (7 Questions)

Standard: Contrast face to chest images (10 Questions)

Standard: Blurry face/ Pixel (13 Questions)

Standard: Timed (5 Questions)

Standard: Ending section (3 Questions)

Standard: Robots in life (4 Questions)

| Page Break |  |
| --- | --- |

Start of Block: Participant information/ Introduction/ Happy Face

Q119 **PARTICIPANT INFORMATION SHEET**  **Study:** A study to investigate the impact of the aesthetics on people’s trust of robots.
 **Project summary** Thank you for participating in this study. This study is designed to examine your thoughts and feelings around different robotic aesthetics. Your participation will enable the collection of data which will form part of a study being undertaken at Cardiff Metropolitan University.
 **Why have you been asked to participate?** You have been asked to participate because the study seeks the opinions of potential end-users of technology and/or robots. Your participation is entirely voluntary, and you may withdraw at any time.
 **Project risk** The research involves the completion of a questionnaire. We are not seeking to collect any sensitive data on you; this study is only concerned with identifying how participants feel about robot aesthetic. We do not think that there are any significant risks associated with this study. However, if you do feel that any of the questions are inappropriate then you can stop at any time. Furthermore, you can change your mind and withdraw from the study at any time - we will completely respect your decision.
 **How we protect your privacy** All the information you provide will be held in confidence. We have taken careful steps to make sure that you cannot be directly identified from the questionnaire form; there is no information on these questionnaires that will identify you. Your personal details (e.g. signature on the consent form) and your questionnaire will be kept in secure locations by the research team (i.e. on the Cardiff met secure OneDrive systems) and all data collected will be anonymized. When we have finished the study and analysed all the information, all the documentation used to gather the data will be destroyed.

Q118

- I Consent (2)

| Page Break |  |
| --- | --- |

Q13 How old are you?

- 15 or under (1)
- 16-21 (3)
- 22-35 (5)
- 36-50 (7)
- 51+ (6)

Q14 What is your gender?

- Male (1)
- Female (2)
- Other (3)

Q17 Do you use (or have had any experience) with robots?

- Yes (1)
- No (2)

Q51 Would you trust a robot in everyday life? (e.g- Your teacher/ Doctor / Receptionist / Driver / Tour guide etc...

- Yes (1)
- No (2)
- Maybe (4)
- Depends on the job (5)

| Page Break |  |
| --- | --- |

Q76 A lot of the questions featured in this survey will surround the topic of trust! Here is the Cambridge definition of the word trust to help you!
 
***"To Believe that someone is good and honest and will not harm you, or that something is safe and reliable"***

 

Q126
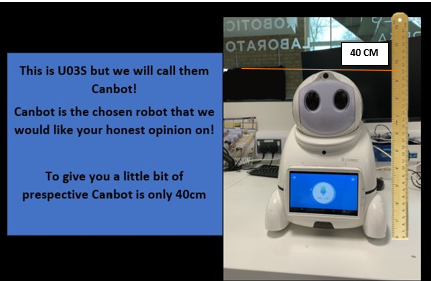


Q55 Would you trust Canbot by the way they look?

- Yes (1)
- Dont know (2)
- No (4)

Q48 In your opinion, how trusting is this robot by their appearance?

|  | 0 | 10 | 20 | 30 | 40 | 50 | 60 | 70 | 80 | 90 | 100 |
| --- | --- | --- | --- | --- | --- | --- | --- | --- | --- | --- | --- |

| 0 = Not very trusting and 100 = very trusting) (1) |  |
| --- | --- |

| Page Break |  |
| --- | --- |

Q109
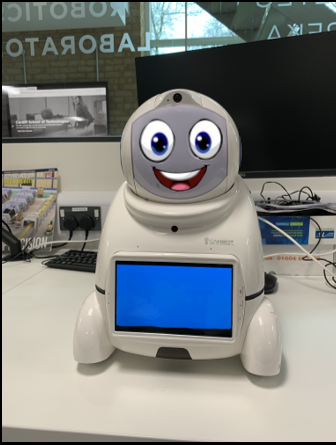


Q57 Do you trust CANBOT more now?

- Yes (1)
- Dont Know (2)
- No (3)

Q112
**_____________________________**

Q110
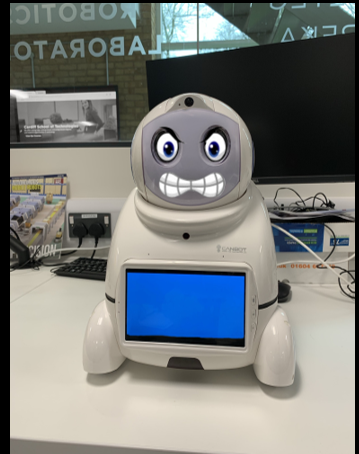


| 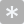 |
| --- |

Q65 What emotion do you think Canbot is feeling? (MAX 2)

- Angry (1)
- Happy (2)
- Sad (3)
- Confused (4)
- Don't know (5)

Q77 Does this affect your ability to trust Canbot?

- More trusting (1)
- About the same (2)
- Less trusting (3)

Q156 Click to write the question text

- Extremely effective (9)
- Very effective (10)
- Moderately effective (11)
- Slightly effective (12)
- Not effective at all (13)

End of Block: Participant information/ Introduction/ Happy Face

Start of Block: Human Eyes

Q111
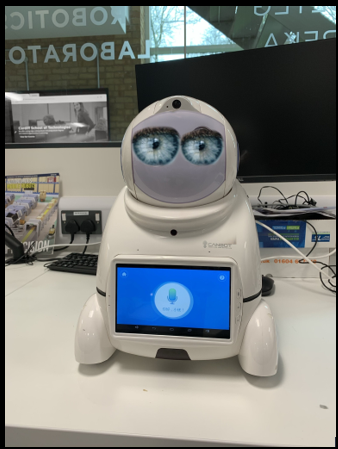


| 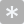 |
| --- |

Q60 How does Canbot make **you** feel with this appearance? ( PICK 3)

- Scared (1)
- Worried (2)
- Happy (3)
- confused (4)
- Excited (5)
- Angry (6)
- Surprised (8)
- Unsure (23)
- I Don't Know (24)

Q62 Do you like Canbot with human eyes?

- Yes (1)
- Don't Know (2)
- No (3)

| Page Break |  |
| --- | --- |

Q90 What do you believe only robots, only humans or both should have? 


Drag features to relevant boxes.

| Robot | Both | Human |
| --- | --- | --- |
| ______ Eyes (1) | ______ Eyes (1) | ______ Eyes (1) |
| ______ Mouth (2) | ______ Mouth (2) | ______ Mouth (2) |
| ______ Nose (3) | ______ Nose (3) | ______ Nose (3) |
| ______ Emotions (4) | ______ Emotions (4) | ______ Emotions (4) |
| ______ Arms/ Legs (5) | ______ Arms/ Legs (5) | ______ Arms/ Legs (5) |
| ______ Feelings (8) | ______ Feelings (8) | ______ Feelings (8) |
| ______ Ability to learn (9) | ______ Ability to learn (9) | ______ Ability to learn (9) |
| ______ To have rights (10) | ______ To have rights (10) | ______ To have rights (10) |
| ______ To be able to work (11) | ______ To be able to work (11) | ______ To be able to work (11) |
| ______ To be trusted (12) | ______ To be trusted (12) | ______ To be trusted (12) |

Q93 Use this space to expand on any answers from above/ Give your reasoning behind them.

________________________________________________________________

End of Block: Human Eyes

Start of Block: Confused face

Q113
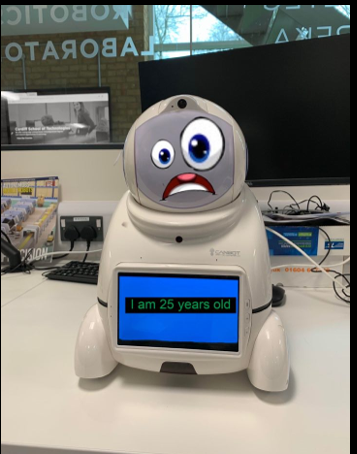


Q70 Do you trust that Canbot is telling you the truth about their age?

- Yes (1)
- Don't Know (2)
- No (3)

Display This Question:

If Q70 = Yes

Q114 You selected you to believe Canbot **is** telling you the truth.
What influence did the facial expression have on your decision?

- A great deal (11)
- A lot (12)
- A moderate amount (13)
- A little (14)
- None at all (15)
- Comments (16) ________________________________________________

Display This Question:

If Q70 = No

Q115 You selected you believe Canbot is **not** telling you the truth.
What influence did the facial expression have on your decision?

- A great deal (11)
- A lot (12)
- A moderate amount (13)
- A little (14)
- None at all (15)
- Comments (16) ________________________________________________

Display This Question:

If Q70 = Don't Know

Q117 You selected you are unsure if Canbot is telling the truth or not.
What influence did the facial expression have on your decision?

- A great deal (30)
- A lot (31)
- A moderate amount (32)
- A little (33)
- None at all (34)
- Comments (22) ________________________________________________

End of Block: Confused face

Start of Block: Colour Changes

Q83 These Canbots have all undergone a slight paint job!


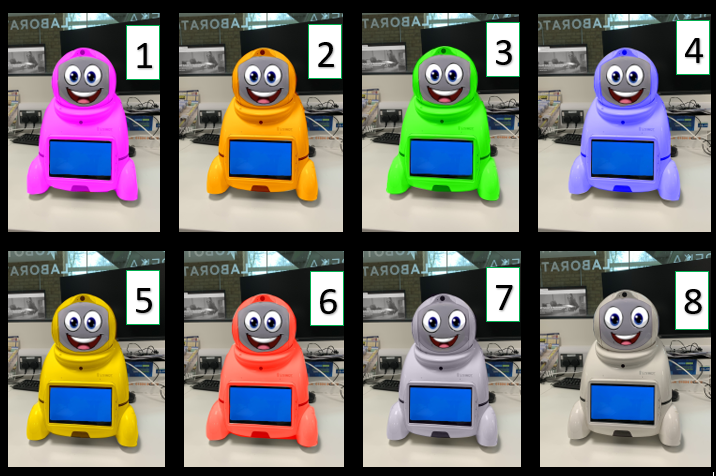


Q84 What robot would you associate with the following words?
(Put the number of the robot 1-8 in the box next to the word)

|  | 1 (1) | 2 (2) | 3 (3) | 4 (4) | 5 (5) | 6 (6) | 7 (7) | 8 (8) | Unsure (9) |
| --- | --- | --- | --- | --- | --- | --- | --- | --- | --- |
| Dangerous (1) |  |  |  |  |  |  |  |  |  |
| Happiest (2) |  |  |  |  |  |  |  |  |  |
| Most trusting (3) |  |  |  |  |  |  |  |  |  |
| Unpredictable (4) |  |  |  |  |  |  |  |  |  |
| Unrealistic (5) |  |  |  |  |  |  |  |  |  |
| Most 'Normal' / Realistic (6) |  |  |  |  |  |  |  |  |  |

Q85 What robot would you trust the most based on the change of colour?

- 1 (1)
- 2 (2)
- 3 (3)
- 4 (4)
- 5 (5)
- 6 (6)
- 7 (7)
- 8 (8)

| Page Break |  |
| --- | --- |

Q87
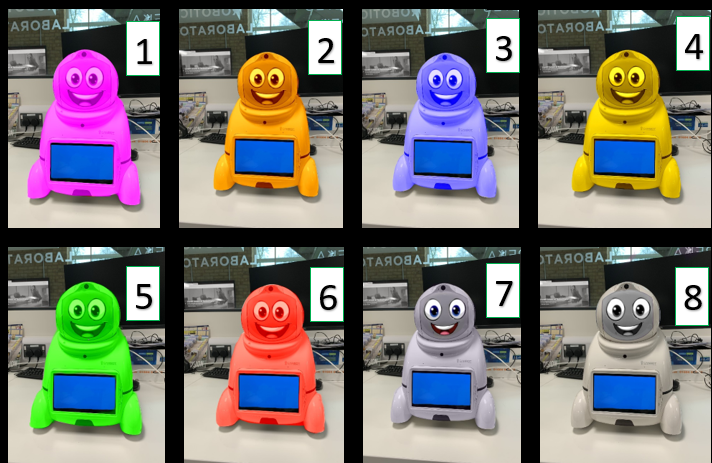


Q105 What robot would you associate with the following words?
(Put the number of the robot 1-8 in the box next to the word)

|  | 1 (1) | 2 (2) | 3 (3) | 4 (4) | 5 (5) | 6 (6) | 7 (7) | 8 (8) | Unsure (9) |
| --- | --- | --- | --- | --- | --- | --- | --- | --- | --- |
| Dangerous (1) |  |  |  |  |  |  |  |  |  |
| Happiest (2) |  |  |  |  |  |  |  |  |  |
| Most trusting (3) |  |  |  |  |  |  |  |  |  |
| Unpredictable (4) |  |  |  |  |  |  |  |  |  |
| Unrealistic (5) |  |  |  |  |  |  |  |  |  |
| Most 'Normal' / Realistic (6) |  |  |  |  |  |  |  |  |  |

Q89 What robot would you trust the most based on the change of colour?

- 1 (1)
- 2 (2)
- 3 (3)
- 4 (4)
- 5 (5)
- 6 (6)
- 7 (7)
- 8 (8)

End of Block: Colour Changes

Start of Block: Tester Pick Red / Contrast images

Q69 PICK RED FROM THE BELOW LIST

- Pink (1)
- blue (2)
- Green (3)
- Red (4)
- Gold (5)
- Silver (7)

| Page Break |  |
| --- | --- |

Q139


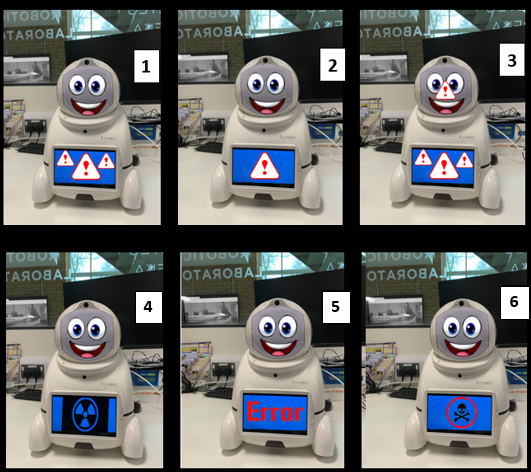


Q140 Rank the above Canbots with the most **untrustworthy** first.
 
(1st - Most untrustworthy, 6th - More trusting)

______ Canbot 1 (1)

______ Canbot 2 (2)

______ Canbot 3 (3)

______ Canbot 4 (4)

______ Canbot 5 (5)

______ Canbot 6 (6)

| Page Break |  |
| --- | --- |

Q153
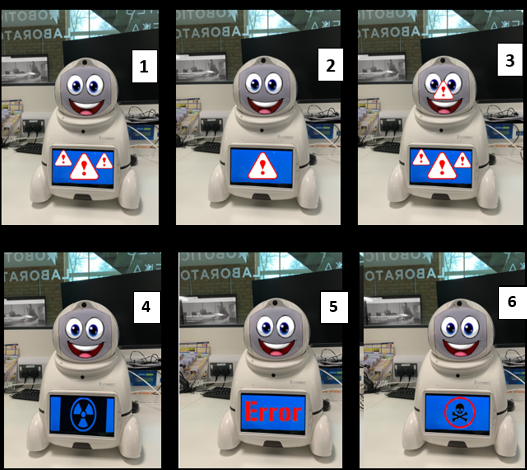


Q142 Rank the above Canbots with the most **Concerning/Scary** first.
 
(1st - Most Concerning/Scary, 6th - Concerning/Scary)

______ Canbot 1 (1)

______ Canbot 2 (2)

______ Canbot 3 (3)

______ Canbot 4 (4)

______ Canbot 5 (5)

______ Canbot 6 (6)

| Page Break |  |
| --- | --- |

Q106
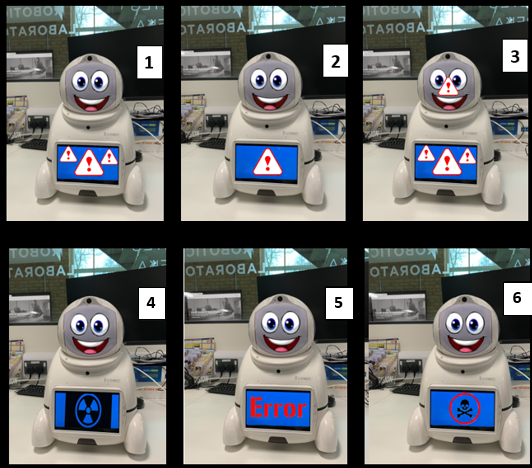


Q107 Write the number of the robot in the end box that you would associate with these words.
(Number 7 for all)
(number 8 for none)

|  | 1 (1) | 2 (2) | 3 (3) | 4 (4) | 5 (5) | 6 (6) | ALL (7) | NONE (8) |
| --- | --- | --- | --- | --- | --- | --- | --- | --- |
| Dangerous (1) |  |  |  |  |  |  |  |  |
| Broken (2) |  |  |  |  |  |  |  |  |
| Happy (3) |  |  |  |  |  |  |  |  |
| Alarming (4) |  |  |  |  |  |  |  |  |
| Scary (5) |  |  |  |  |  |  |  |  |
| Concerning (6) |  |  |  |  |  |  |  |  |
| Untrustworthy (7) |  |  |  |  |  |  |  |  |
| Confusing (8) |  |  |  |  |  |  |  |  |

End of Block: Tester Pick Red / Contrast images

Start of Block: Contrast face to chest images

Q127
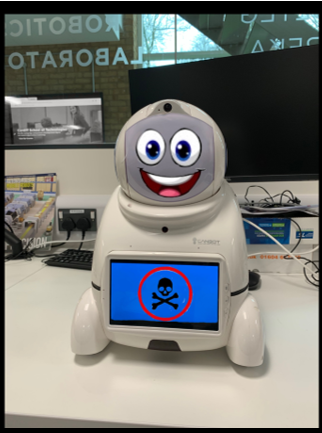


Q128 What emotion do you believe **Canbot** feels?

- Happy (1)
- Sad (2)
- Angry (3)
- Surprised (4)
- Excited (5)
- Confused (6)
- Tired (7)

Q129 Do you trust Canbot here

- Yes (1)
- No (2)
- Not sure (4)

| Page Break |  |
| --- | --- |

Q131
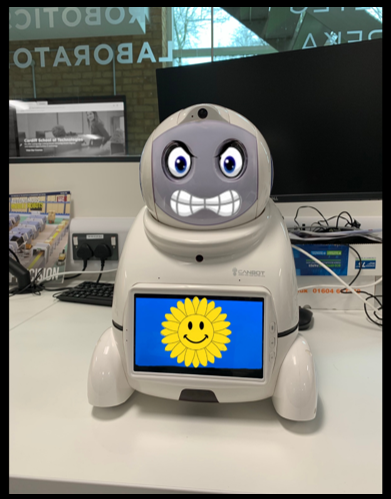


Q132 What emotion do you believe **Canbot** feels?

- Happy (1)
- Sad (2)
- Angry (3)
- Surprised (4)
- Excited (5)
- Confused (6)
- Tired (7)

Q133 Do you trust Canbot here

- Yes (1)
- No (2)
- Not sure (4)

| Page Break |  |
| --- | --- |

Q135
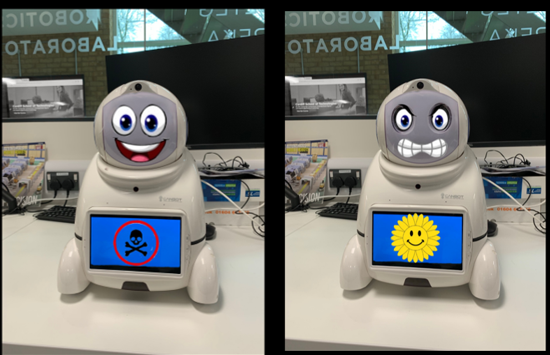


Q136 Rank the order of what first caught your attention on these two images.
(1st being the first thing to catch your attention)

______ Happy Face (1)

______ Angry Face (4)

______ Sun icon (5)

______ Danger Icon (6)

______ Something else (3)

Display This Question:

If Q136 [ ] > 3

Q137 What else did you first notice from these images?

________________________________________________________________

Q138 Does the facial expression overrule the icon on the chest screen when considering Canbots emotions?

- Yes (1)
- No (2)
- Don't Know/ Unsure (4)

End of Block: Contrast face to chest images

Start of Block: Blurry face/ Pixel

Q124
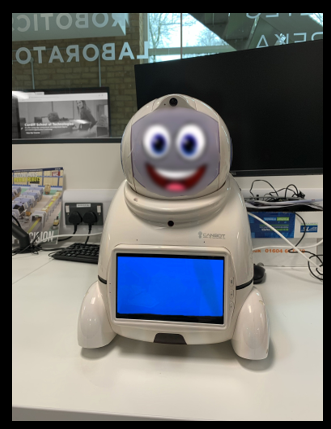


| 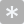 |
| --- |

Q73 How does this Canbot make **you** feel?  (MAX 3)

- Uncertain (7)
- Trusting (6)
- Uneasy (8)
- Happy (5)
- Uncomfortable (9)
- Sad (10)
- Confused (4)
- Angry (11)
- Disgust (12)
- No change/ Unsure (13)
- Other (14) ________________________________________________

Q74 What emotion do you think **Canbot** feels?

- Uncertain (7)
- Happy (1)
- Sad (2)
- Disgusted (9)
- Uncomfortable (8)
- Angry (3)
- Confused (4)
- Uneasy (10)
- Don't know (5)
- Other (6) ________________________________________________

| Page Break |  |
| --- | --- |

Q143
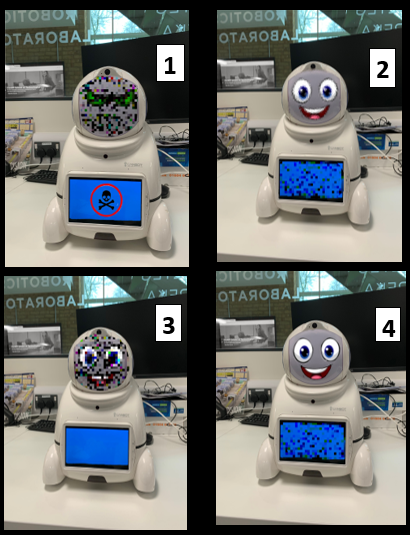


Q144 What Canbot would you describe as the most **uncertain/Confusing**?


(1 -Most uncertain/Confusing , 4 - More certain/ Less confusing)

______ Canbot 1 (1)

______ Canbot 2 (2)

______ Canbot 3 (3)

______ Canbot 4 (4)

| Page Break |  |
| --- | --- |

Q155
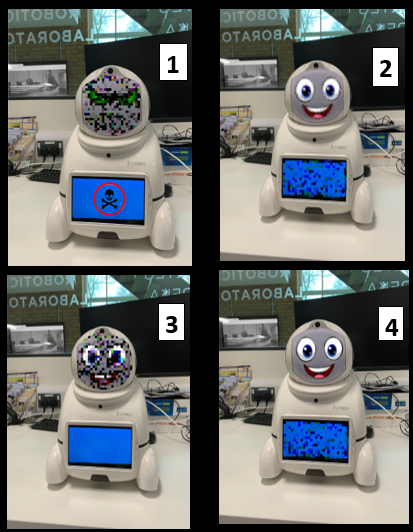


Q145 What Canbot would you describe as most **trusting**?
 

 (1- Most trusting  4- Least trusting)

______ Canbot 1 (1)

______ Canbot 2 (2)

______ Canbot 3 (3)

______ Canbot 4 (4)

| Page Break |  |
| --- | --- |


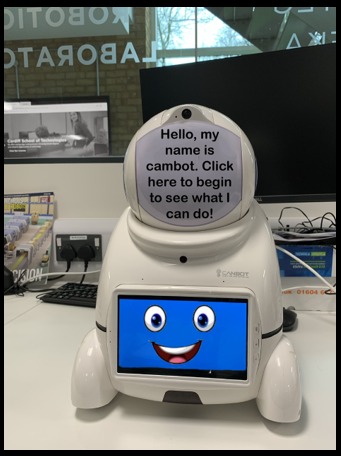
Q148

Q149 Would you trust this Canbot?

- Yes (1)
- No (2)
- Don't Know (4)

Display This Question:

If Q149 = Yes

Q150 You selected you would **trust** Canbot!
 Did the fact the face was in the wrong location have any impact on your decision?

- Yes (1)
- No (2)
- Don't Know (3)

Display This Question:

If Q149 = No

Q152 You selected you would not trust Canbot!
Did the fact the face was in the wrong location have any impact on your decision?

- Yes (1)
- No (2)
- Don't Know (3)

| Page Break |  |
| --- | --- |

Q146


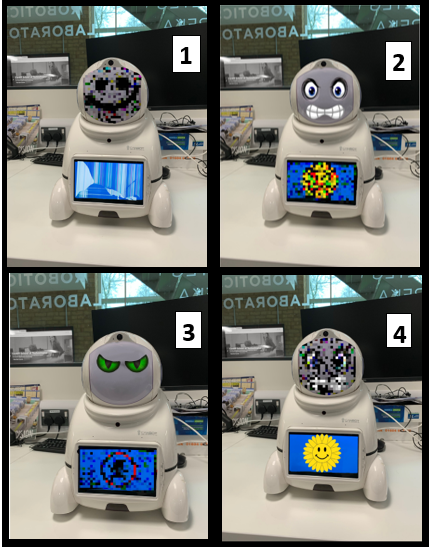


Q147 Rank the robots in order of most trusting.
(1- Most trusting 4- Least trusting)

______ Canbot 1 (1)

______ Canbot 2 (2)

______ Canbot 3 (3)

______ Canbot 4 (4)

End of Block: Blurry face/ Pixel

Start of Block: Timed

Q78 The following questions are timed! The duration will be recorded and monitored. 

 
The following question we will show you a maths equation and one of the robots has the correct answer. Click the robot with what you believe to be the solution to the problem. 
 
Please note we are not testing your mathematical ability just your willingness to trust one of the robots, **Do not** use a calculator to work out the correct answer.

| Page Break |  |
| --- | --- |

Q82 What robot do you believe is giving you the correct answer to the below equation?
 (NO CALCULATOR)
  
 997 x 1066 =
Click the number (1-5) or the robot
 Then click the next arrow


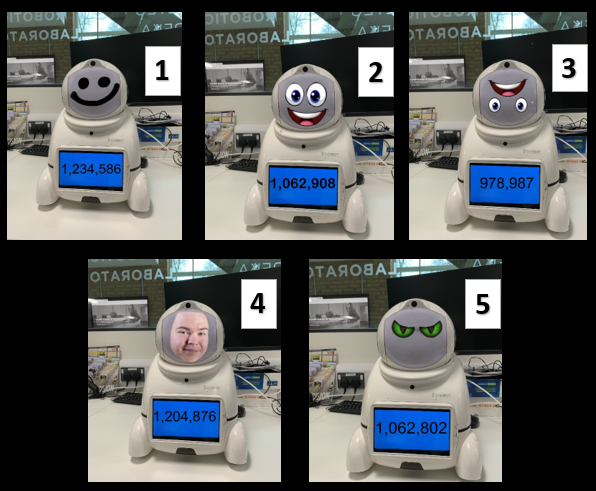


Q125 Timing

First Click (1)

Last Click (2)

Page Submit (3)

Click Count (4)

| Page Break |  |
| --- | --- |

Q91 What robot do you believe is giving you the correct answer to the below equation?
 (NO CALCULATOR)
  

877 x 974 =
Click the number (1-5) or the robot
Then click the next arrow


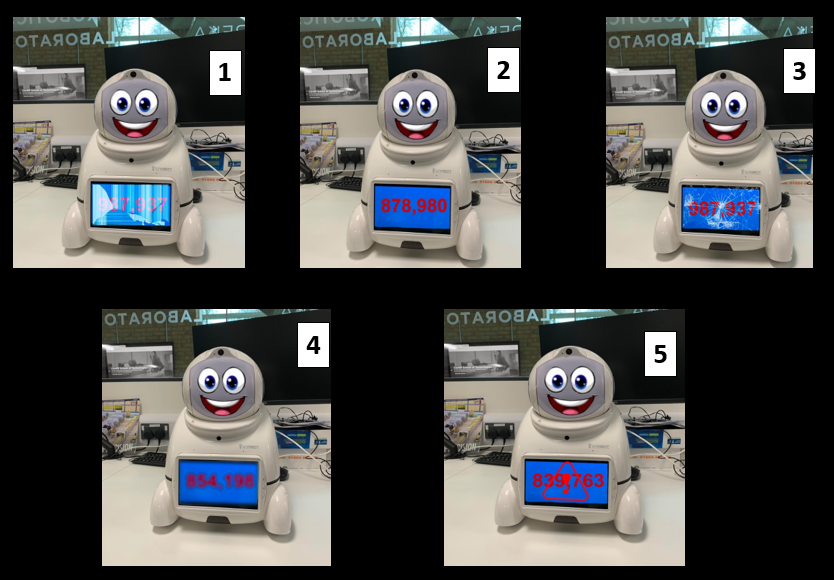


Q92 Timing

First Click (1)

Last Click (2)

Page Submit (3)

Click Count (4)

End of Block: Timed

Start of Block: Ending section

Q25 Have you any thoughts on how you would design a robot that people would trust?

________________________________________________________________

________________________________________________________________

________________________________________________________________

________________________________________________________________

________________________________________________________________

Q67 How important do you feel that the below factors are to build trust?

|  | Very Important (1) | Important (2) | Neutral (3) | Unimportant (4) | Not at all important (5) |
| --- | --- | --- | --- | --- | --- |
| Show commitment (4) |  |  |  |  |  |
| Leads by example (5) |  |  |  |  |  |
| Caring (6) |  |  |  |  |  |
| Ability to listen (7) |  |  |  |  |  |
| Ability to keep secrets (8) |  |  |  |  |  |
| Honest (9) |  |  |  |  |  |
| Respects others (10) |  |  |  |  |  |
| Be a friend (11) |  |  |  |  |  |
| Be fair (12) |  |  |  |  |  |

Q68 How much does the colour of a product affect your decision making?

|  | 0 | 10 | 20 | 30 | 40 | 50 | 60 | 70 | 80 | 90 | 100 |
| --- | --- | --- | --- | --- | --- | --- | --- | --- | --- | --- | --- |

| 0- Not at all /100- A lot (1) |  |
| --- | --- |

End of Block: Ending section

Start of Block: Robots in life

Q94 In this question we are interested in what jobs you would trust a robot to do. 
Simply click the job and select the relevant answer from the options.

|  | Robot (1) | Would not mind both (2) | Human (3) |
| --- | --- | --- | --- |
| 1: Doctor (58) |  |  |  |
| 2: Driver (59) |  |  |  |
| 3: Dog-walker (60) |  |  |  |
| 4: Shop-assistant (61) |  |  |  |
| 5: Office-worker (62) |  |  |  |
| 6: Surgeon (63) |  |  |  |
| 7: Military (64) |  |  |  |
| 8: Teacher (65) |  |  |  |
| 9: Accountant (66) |  |  |  |
| 10: Actor (67) |  |  |  |
| 11: Pilot (68) |  |  |  |
| 12: Police-officer (69) |  |  |  |

| Page Break |  |
| --- | --- |

Q97 How often do you see a robot in everyday life?

- Daily (7)
- Weekly (11)
- Montly (12)
- Every 6-Months (13)
- Yearly (14)
- Over a year ago (15)
- Never (10)

Q98 "I am nervous that a robot might one day take my job"

- Strongly agree (4)
- Agree (5)
- Somewhat agree (6)
- Neither agree nor disagree (7)
- Somewhat disagree (8)
- Disagree (9)
- Strongly disagree (10)
- My job cannot be done by a robot! (11)

Display This Question:

If Q98 = My job cannot be done by a robot!

Q99 You have selected your job cannot be done by robots, why is this?

________________________________________________________________

End of Block: Robots in life
